# Aggregation Behaviour as an Adaptive Reproductive Strategy in a Marine Ecosystem Engineer

**DOI:** 10.1002/ece3.71413

**Published:** 2025-05-20

**Authors:** Marine Uguen, Sylvie M. Gaudron, Alexandre Rahoui‐Davoust, Valérie Lefebvre, Laurent Seuront

**Affiliations:** ^1^ Université de Lille, CNRS, Université du Littoral Côte D'opale, IRD, UMR Laboratoire D'océanologie et de Géosciences Lille France; ^2^ Sorbonne Université Paris France; ^3^ English Channel and North Sea Research Unit IFREMER Boulogne‐sur‐Mer France; ^4^ Department of Zoology and Entomology Rhodes University Grahamstown South Africa; ^5^ Department of Marine Resources and Energy Tokyo University of Marine Science and Technology Tokyo Japan

**Keywords:** gregarious behaviour, group dynamic, locomotion, marine invertebrates, movement, reproduction

## Abstract

Aggregation behaviour is a key process shared by numerous organisms, providing benefits such as predator protection, resistance to abiotic stressors and enhancing reproductive success. In coastal waters, mussel aggregations shape ecosystems and increase biodiversity; however, many aspects of this ecologically key behaviour remain unexplored. Here, we investigated the potential link between aggregation rate and reproductive maturity stages in the blue mussel *Mytilus edulis* over a seasonal cycle. We showed that the seasonality of *M. edulis* aggregation rate was a significant sinusoidal function of the mean reproductive maturity stage. Specifically, aggregation increased during gamete maturation until the onset of spawning and subsequently declined, following a cyclical pattern, supporting a reproductive function of this aggregation behaviour in mussels. Given the ubiquity of plastics as marine contaminants and their known harmful effects on organisms, we subsequently assessed how the temporal patterns observed in aggregation behaviour may be impacted by an exposure to leachates from plastic pellets. Thus, an exposure to plastic leachates led to a loss of the cyclical pattern observed in control seawater, potentially compromising mussel reproduction and the stability of mussel bed habitat. Our study showed the relationship between aggregation behaviour, reproduction and contaminants in mussels. Understanding these complex interactions is crucial, given the pivotal role mussels and their aggregations play in shaping marine ecosystems, offering new insights into the resilience of this habitat facing contemporary challenges.

## Graphical abstract

Mussels play a key role in coastal ecosystems by forming dense aggregations that provide habitats and enhance biodiversity. Here, we showed that the mussel aggregation rate varies seasonally according to their reproductive cycle, increasing during gamete maturation and declining postspawning, supporting a reproductive function for this behaviour. In this context, we subsequently found that exposure to plastic contaminants disrupts this seasonal cycle, potentially threatening their reproductive success, mussel bed stability and associated ecosystem services.

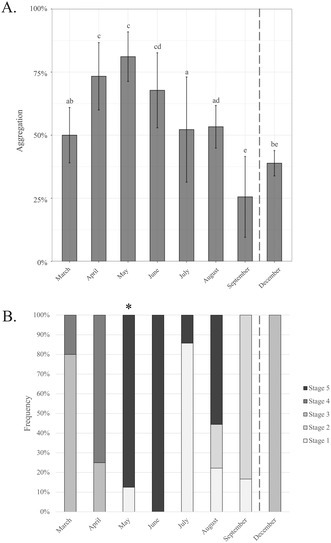

## 1 Introduction

Aggregation behaviour, that is, the tendency of animals to group together, is a fundamental ecological process, which confers important benefits on individual fitness, such as reducing the predation risk, dampening the abiotic stressors and providing better access to resources and mates (Allee 1931; Parrish and Edelstein‐Keshet 1999). The benefits of aggregation may extend well beyond the individual level, as group formation may lead to the completion and maintenance of the entire ecosystem. Specifically, in the marine environment, the aggregations of certain species of bivalves, corals or sponges generate complex three‐dimensional biogenic reef structures, often referred to as ‘marine animal forests’ (Rossi et al. 2017). These structures provide new habitats and alleviate physical stress, which often leads to changes in species assemblages, modification of the structure of the food web and favour the establishment and sustainability of biodiversity hotspots (Jones et al. 1997; Romero et al. 2015).

Some of these engineer species, such as corals and sponges, typically aggregate during their settlement and subsequently do not mostly move during their adult life. In contrast, mussels do not lose their ability to move during their juvenile and adult stages after larval settlement. Instead, they use their foot and byssal gland complex to move, readjust their position and firmly attach themselves to the substrate and form dynamic aggregations referred to as mussel beds (Commito et al. 2014; Seuront et al. 2021; Zardi et al. 2021). Beyond offering an optimal habitat to a wide range of species (Borthagaray and Carranza 2007), these aggregations protect mussels from predation (Côté and Jelnikar 1999; Nicastro et al. 2007; Reimer and Tedengren 1997), wave dislodgment (Zardi et al. 2021) and fluctuations in temperature and humidity (Nicastro et al. 2012). Aggregation behaviour has also been shown to be a reproductive strategy in numerous benthic broadcast spawners (e.g., Himmelman et al. 2008; Keesing et al. 2011; Marquet et al. 2018; Mendo et al. 2014; Shepherd 1986; Simon and Levitan 2011). In broadcast spawners, such as mussels, the population density and distance between mates are commonly referred to as among the most important processes in fertilisation success (Claereboudt 1999; Levitan and Petersen 1995; Liu et al. 2011; McEdward 1995; Molloy et al. 2012), especially given gamete dilution and that contact time between gametes required for successful fertilisation is only a few minutes in mussels (Liu 2009). Beyond these proximity benefits, field and laboratory experiments showed that in mussels, patch formation creates empty spaces between aggregates, generating recirculation zones that reduce sperm dilution and enhance sperm–oocytes encounters, ultimately increasing fertilisation success (Quinn and Ackerman 2011). As previously suggested for another broadcast spawner, the limpet *Patella vulgata* (Coleman et al. 2006), considering aggregation behaviour as a reproductive strategy would result in an increase in the tendency to aggregate when gametogenesis is completed. In this context, we hypothesise that mussel aggregation behaviour acts as a reproductive strategy, with aggregation increasing with gametogenesis in order to enhance the fertilisation success.

However, the behavioural responses of marine organisms, including mussels, can be impaired by a wide range of anthropogenic contaminants present in the marine environment (Melvin and Wilson 2013). Among them, plastic is the most common marine contaminant, with 4.8–12.7 million tonnes estimated to be released into the ocean each year (Jambeck et al. 2015). Plastics are associated with numerous chemical compounds such as additives (e.g., phthalates, bisphenol A, and antioxidants) that can be leached out once in the environment and affect marine organisms; see Delaeter et al. (2022) for a review. Specifically, plastic leachates have been shown to disrupt the aggregation, locomotion, chemical perception and reproductive success of mussels (see Uguen et al. 2025 for a review). Despite the range of observed responses following exposure to plastic leachates, there is still a lack of information on how plastic pollution may impact mussel aggregation behaviour in particular over the course of a seasonal cycle.

Here, we chose the blue mussel *Mytilus edulis* as a model organism. This gonochoric species exhibits a seasonal reproduction, typically characterised by one or two spawning events usually triggered by an increase in temperature above 10°C–12°C when gametogenesis is completed (Boromtharanat et al. 1987). Despite the plethora of research devoted to the reproduction of this species in the Atlantic Ocean since the mid‐20th century (Lubet 1959), there is still a critical lack of knowledge on *M. edulis* reproduction in the Eastern English Channel (EEC), where they hold economic, social and patrimonial values (Dauvin 2019). Moreover, the EEC is heavily impacted by various marine and land‐based types of pollution (Dauvin 2019; Tappin and Millward 2015), including plastic (Gravier and Haut 2020). For instance, in 2016, 8 tonnes of plastic pellets were spilt from a tanker truck on a highway located *ca*. 2.5 km from the shoreline, dispersed through the surrounding waterways, leading to significant shoreline contamination that persisted for several years after the accident (Gravier and Haut 2020). However, the biological and ecological effects of plastic pollution in the EEC are still relatively unknown, despite recent efforts to bridge this knowledge gap (Seuront 2018; Seuront et al. 2021; Uguen et al. 2022, 2023, 2024; Zardi et al. 2024a, 2024b).

In this context, the aim of this paper was first to test the hypothesis that the aggregation behaviour of *M. edulis* can be considered a reproductive strategy, in which case we would expect an increase in the tendency to aggregate when gametogenesis is completed. To do so, we combined behavioural assays and histological analyses over an 8‐month study. Through an additional treatment, we subsequently assessed whether exposure to leachates of commercially available polypropylene pellets may impair the observed seasonal patterns in aggregation behaviour.

## 2 Material and Methods

### 2.1 Sampling

*Mytilus edulis* adult individuals (shell length: 2.5–3.5 cm, *n* = 200) were sampled monthly from March to December 2021 (with the exception of October and November 2021) from a rocky intertidal reef (Pointe aux Oies, Wimereux; 50°47′08.3″ N, 1°36′03.9″ E) located along the French coasts of the eastern English Channel. Mussels (*n* = 180) were acclimated for 24 h in the laboratory in 85‐L tanks filled with running aerated natural seawater representative of in situ conditions of temperature and salinity (Appendix S1) before the behavioural assays took place. Concurrently, for the histological analysis, mussels (*n* = 20) were dissected and each gonad was fixed in 10% formaldehyde and stored at 4°C. As *M. edulis* is a gonochoric species and gametes spread outside of the gonad to the mantle lobes during the completion of gametogenesis (Seed 1969), to determine the gender (male/female) of each specimen, a biopsy of this mantle was undertaken and observed under a microscope (Motic BA210) during the dissection.

### 2.2 Microplastic Leachate Solution

Mussels were exposed to either (i) control natural seawater or (ii) microplastic leachate solution (MPL). MPL was prepared from commercially available polypropylene pellets (3.80 ± 0.21 × 2.95 ± 0.50 mm; translucent white; Pemmiproducts, Aachen, Germany) incubated for 24 h at 12°C in natural aerated seawater at a concentration of 20 mL of pellets (*ca*. 12 g; 400 pellets) per litre. The pellet incubation temperature was fixed at 12°C during the 8‐month study to avoid any biases related to different desorption kinetics of additives due to temperature (e.g., Kida et al. 2022; Kida and Koszelnik 2021).

The additives contained in the polypropylene pellets before and after their 24 h incubation were analysed in a previous study (Uguen et al. 2023) using a pyrolysis analysis coupled with gas chromatography and a high‐resolution spectrometer (Appendix S2). A total of 27 additives were identified, including three brominated flame retardants, six phosphorus flame retardants, five antioxidants and 13 plasticisers (see Appendix S2 for further details).

### 2.3 Aggregation Behaviour Assessment

Mussels (*n =* 15) were placed in a 22 cm glass arena, their narrow end facing the centre of the arena in two concentric circles, at *ca*. 1 body length from each other. The arenas were filled with 1.5 L of control seawater (*N =* 6) or MPL (*N =* 6). Mussel position was recorded every minute for 2 h using a GoPro camera (GoPro HERO7 Black, GoPro Inc., San Mateo, California, USA; Uguen et al. 2023; Videos S3, S4). To minimise stress, each mussel was only used once, and byssal threads were carefully cut prior to the experiment. Experiments were conducted in the absence of any acoustic or mechanical disturbances in a temperature‐controlled room. To prevent stress from abrupt temperature shifts and to maintain mussels in their natural physiological state, the temperature of the room was set to the mean temperature recorded in situ (where mussels were collected) ranging from a minimum of 8°C in March to a maximum of 19°C in September (Appendix S1). At the end of the experiment, aggregation behaviour was quantified as the proportion of mussels (%) forming aggregates defined as two or more mussels with their shells in direct physical contact (Commito et al. 2014, 2016; Reimer and Tedengren 1997; Seuront et al. 2021; Uguen et al. 2023). The number of individuals per aggregate was also considered to refine our understanding of the nature of the overall aggregation process.

### 2.4 Reproductive Maturity Stages Assessment

Gonads were dehydrated in an ethanol series (70%–100%), cleared in Diasolv and impregnated in paraffin. Each sample was then sectioned at 5 μm using a microtome (Leica Ltd.) and stained with Haematoxylin and Eosin (Mammone et al. 2020). Histological sections were examined under a microscope (Axioscope 5, Zeiss Ltd.) equipped with the software Zen (Zeiss Ltd.).

Specimen reproductive maturity stages of both males and females were assessed based on criteria established in previous studies (Appendix S5). Specifically, the reproductive maturity for each individual was divided into five stages (*x*_*i*_) ranked from 1 to 5: undifferentiated (*x*_1_), early development (*x*_2_), late development (*x*_3_), ripe (*x*_4_) and partially spent (*x*_5_). In addition, the mean reproductive maturity stage (*μ*) was calculated for each month by weighting each stage rank by its frequency and then dividing by the total number of individuals, as:

$$\mu =\frac{1}{N}\sum_{i=1}^{5}n_{i}\cdot x_{i}\;$$

where x_i is the_ *n*_*i*_ is the number of individuals in the development stage *x*_*i*_, and *N* is the total number of individuals (Choi et al. 2022; Appendix S5).

### 2.5 Statistical Analyses

To assess seasonal variation in mussel aggregation behaviour, a Kruskal–Wallis test (KW test hereafter) was performed for each control and MPL treatment with time as factors (eight levels: March, April, May, June, July, August, September and December) and aggregation behaviour as the dependent variable. The KW test was followed by the powerful Conover–Iman test with Holm's correction to identify distinct groups of measurements; the Conover–Iman test (Conover test hereafter) was used instead of the less powerful though more well‐known Dunn test (Gilbert 2019). To assess the presence of a cyclic relationship between mussel aggregation and reproductive stage, the aggregation rate (A(*μ*); %) observed in the control seawater was expressed as a function of the mean reproductive stage (*μ*), and fitted with a sinusoid of the form:

$$A\left(\mu \right)=a\cdot \sin\;(\omega \text{·μ}+\phi \text{)}+b$$

where the amplitude (*a*), the angular frequency (*𝜔*), the phase shift (*𝜙*) and the baseline (*b*) of the sinusoidal wave were estimated using a nonlinear least‐squares method. This method aimed to minimise the sum of squares residual (SSR) and maximise the coefficient of determination *R* ^2^. To assess the impact of MPL on this cyclic pattern, the proportion of aggregated mussels in MPL was compared with the nonlinear sinusoid equation fitted for control data by measuring the SSR and *R* ^2^.

## 3 Results

### 3.1 *Mytilus edulis* Aggregation Behaviour

**FIGURE 1:**
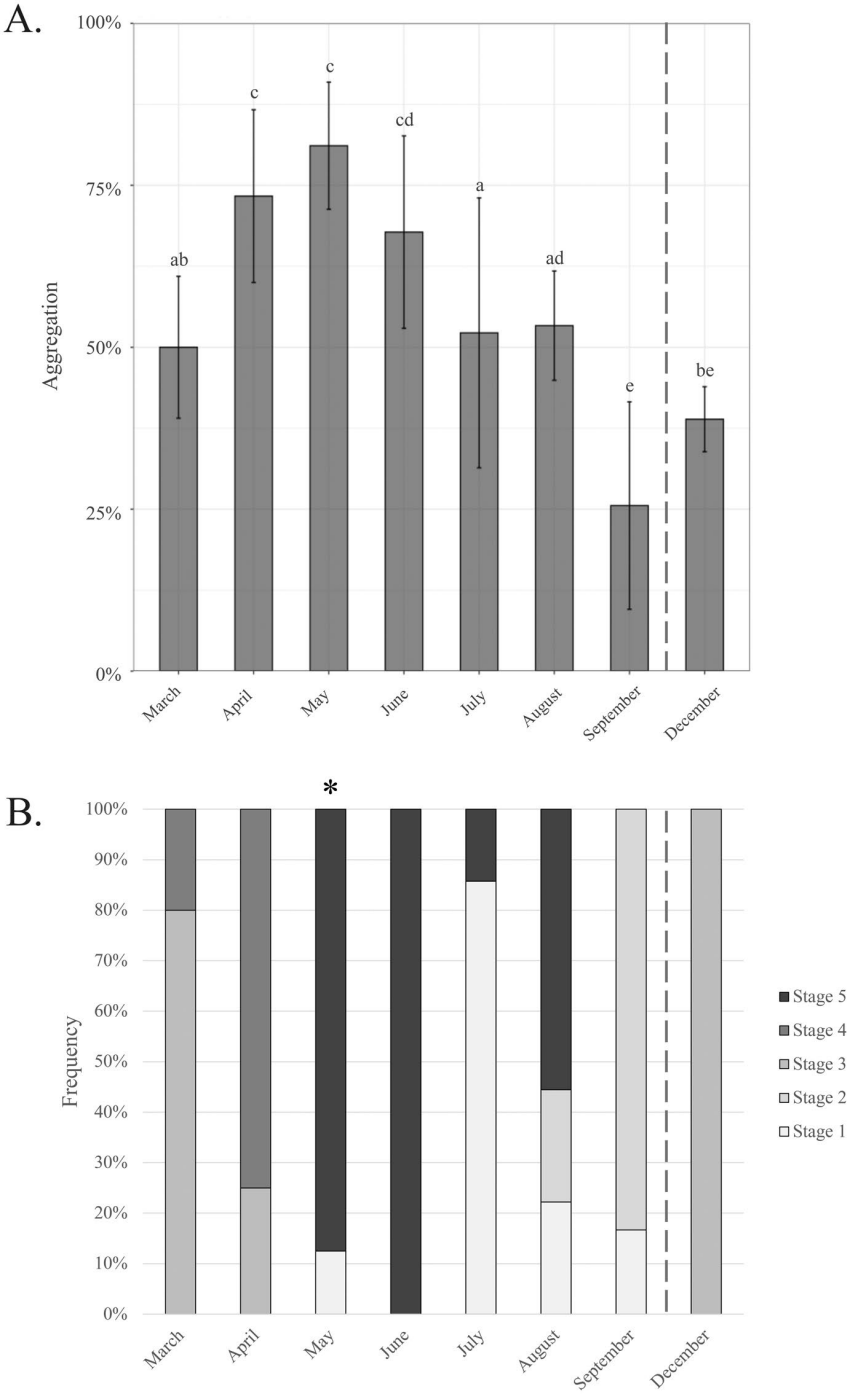
Monthly variation of *Mytilus edulis* (A) aggregation (expressed as the proportion of aggregated individuals; %) at the end of the 2‐h experiment (mean ± standard deviation) and (B) reproductive maturity stages, that is, undifferentiated (Stage 1), early development (Stage 2), late development (Stage 3), ripe (Stage 4) and partially spent (Stage 5). Dashed line indicates a 2‐month time gap with no sampling. Letters depict significant differences (*p* < 0.05; Conover test; Appendix S6) among treatments. Asterisks indicate the start of the spawning period.

**FIGURE 2:**
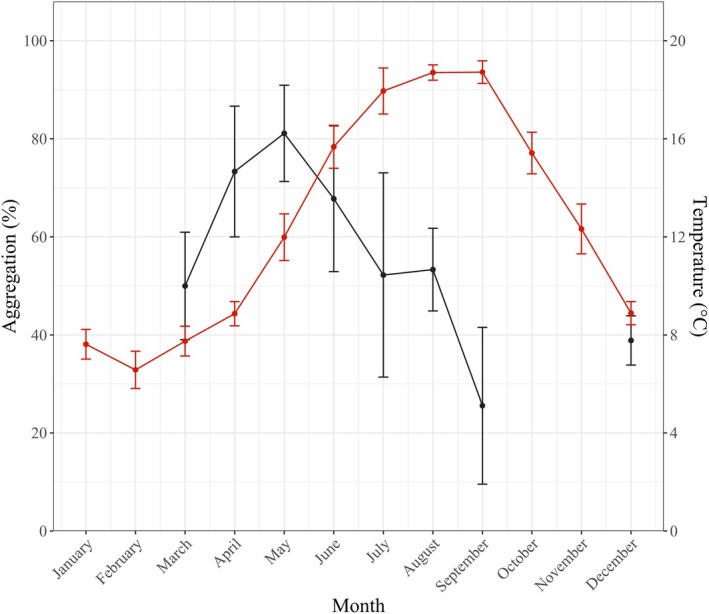
Monthly variation in *Mytilus edulis* aggregation (expressed as the proportion of aggregated individuals; %) at the end of the 2‐h experiment (mean ± standard deviation; black line) and in situ sea surface temperature in 2021 at the study location (mean ± standard deviation; red line), which corresponds to the temperature used in the experiment. Temperature data were obtained from the Coastal Coriolis data portal, for the MAREL Carnot station point (50°74′05″ N, 1°56′77″ E; https://data.coriolis‐cotier.org).

In control seawater, time had a significant effect on the blue mussel *M. edulis* aggregation behaviour (KW test, H = 31.363, df = 7, *p* < 0.001; Figure 1A). Specifically, the aggregation rate increased significantly until it reached a maximum in May (i.e., 81.1% ± 9.8%; mean ± standard deviation, SD) and subsequently decreased significantly until a minimum in September (25.6% ± 16.0%), which was noticeably 3.2‐fold lower than in May. The aggregation rate remained low in December (38.9% ± 5.0%) and was not significantly different from September (see Appendix S6 for the exact Conover *p* values). Note that mussel aggregations were not only more frequent in May but also larger in size; in May, 27% of the aggregations were composed of only two individuals, whereas in September, this proportion increased to 77%. It is finally stressed that the observed aggregation pattern is independent of seawater temperature seasonality, as no correlation was found between aggregation and temperature (Spearman correlation, *p* > 0.05; Figure 2).

### 3.2 *Mytilus edulis* Reproductive Maturity

Reproductive maturity stages typically varied with time (Figure 1B), with mussels in a prespawning condition before May, and where, from March to April, gametogenesis for both genders was completed with fully grown oocytes for females and the presence of spermatozoids in rosettes for males (Stage 4, ripe; Figure 1B; Appendix S5). May marked the onset of the main spawning period (i.e., characterised by the appearance of partially spent specimens; Stage 5; Figure 1B; Appendix S5) which lasted for some mussels until August, when a second minor spawning event may have occurred. In some cases, specimens were completely empty already in July (Figure 1B; Stage 1; Appendix S5). In September, both male and female mussels were mostly devoid of mature gametes (or some atretic) and started a new cycle of reproductive development (Stages 1 and 2; Figure 1B; Appendix S5). Finally, in December, females carried oocytes in vitellogenesis and males mostly spermatocytes (Stage 3; Figure 1B; Appendix S5).

### 3.3 Aggregation Behaviour and Reproductive Maturity

**FIGURE 3:**
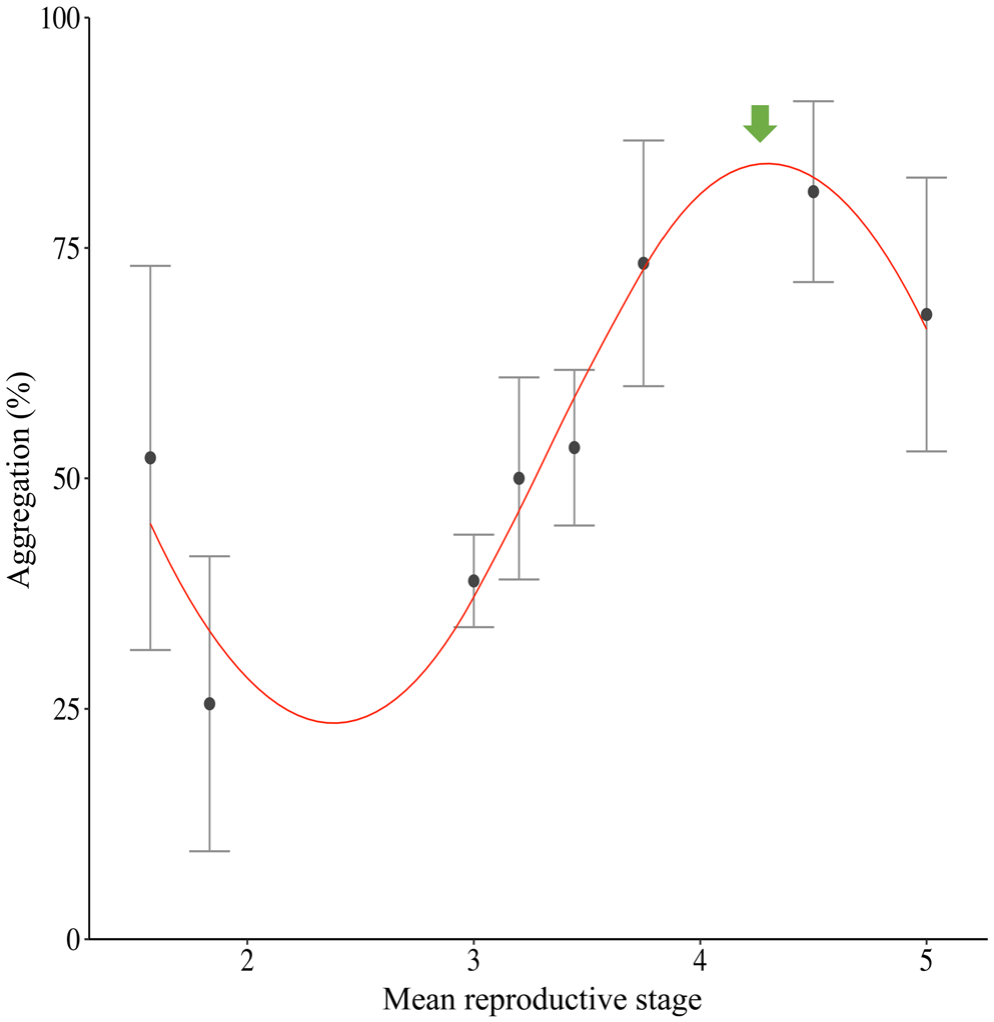
Relationship between the aggregation (expressed as the proportion of aggregated individuals; %) in *Mytilus edulis* at the end of the 2‐h experiment (mean ± standard deviation) and its mean reproductive maturity stages undifferentiated (Stage 1), early development (Stage 2), late development (Stage 3), ripe (Stage 4) and partially spent (Stage 5). Red line represents the best sinusoidal fit between the aggregation (*A*) and the mean reproductive stage (*μ*): A(*μ*) = 31.04 · (1.63 · *μ*–11.72) + 53.28. The green arrow indicates the onset of the spawning period.

*Mytilus edulis* aggregation behaviour varied cyclically according to its reproductive maturity stages (Figure 3). The relationship between mussel aggregation and reproductive stage was consistently highly significantly described by the sinusoidal equation: A(*μ*) = 31.04 · *sin* (1.63 · *μ*—11.72) + 53.28 (SSR = 163; *R* ^2^ = 0.965; *p* < 0.001). Specifically, aggregation increased with reproductive maturity until the start of spawning and then decreased until mussels entered a new reproductive cycle.

### 3.4 Microplastic Leachates and Aggregation Pattern

**FIGURE 4:**
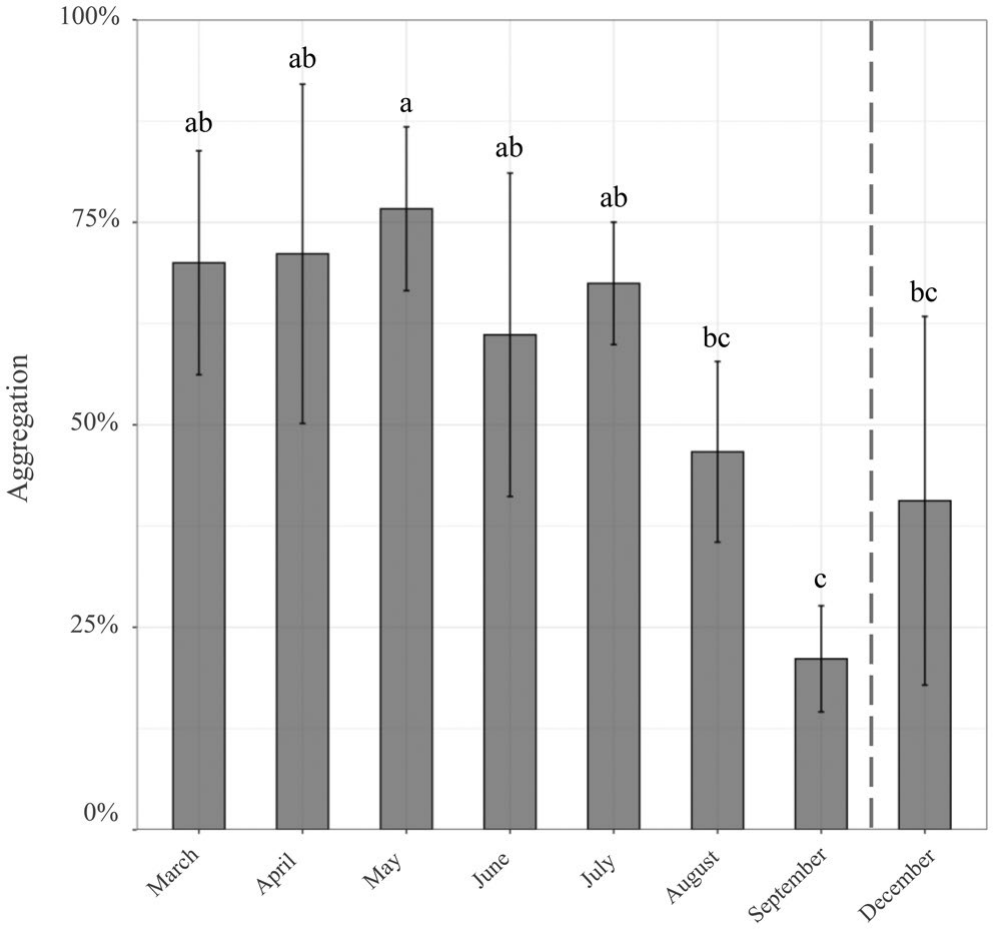
Monthly variation of the aggregation (expressed as the proportion of aggregated individuals; %) in *Mytilus edulis* after exposure to microplastic leachate solution at the end of the 2‐h experiment (mean ± standard deviation). Dashed line indicates a 2‐month time gap with no sampling. Letters depict significant differences (*p* < 0.05; Conover test) among treatments.

When exposed to MPL, time had a significant effect on the aggregation behaviour of *M. edulis* (KW test, *H* = 26.919, df = 7, *p* < 0.001; Figure 4). However, this temporal pattern significantly differed from observations conducted in control seawater, especially with a lack of the peak in aggregation behaviour in May at the onset of the spawning period (Figure 4). Mussel aggregation was high and relatively constant from March to July (with a minimum of 61.1% ± 20.0% in June and a maximum of 76.7% ± 10.1% in May), before significantly decreasing until a minimum in September (21.1% ± 6.5%) and remaining low in December (40.6% ± 22.7%; see Appendix S7, for the exact Conover *p* values).

The cyclic pattern that linked mussel aggregation to reproductive maturity stages in the control was noticeably lost following MPL exposure. Indeed, when exposed to MPL, *M. edulis* aggregation could not be significantly described by the previous sinusoidal function of the mean reproductive stage found in control seawater (*p* > 0.05; Appendix S8).

## 4 Discussion

### 4.1 Aggregation Behaviour as a Reproductive Strategy?

Our results show that *Mytilus edulis* aggregation behaviour was described as a sinusoidal function of its reproductive maturity stages, with aggregation levels consistently the highest when ripe gametes occurred, hence when the spawning event approached. This pattern may indicate that aggregation behaviour—typically considered as an adaptive strategy to minimise predation and wave dislodgement risks (e.g., Côté and Jelnikar 1999; Nicastro et al. 2007; Zardi et al. 2021)—may also play a role in the reproduction of this species. It has been shown in previous studies that the spatial organisation of mussels in aggregates enhanced their fertilisation success (Claereboudt 1999; Quinn and Ackerman 2011). In this context, the fact that *M. edulis* aggregation is not constant throughout the year but varies seasonally according to its reproductive state (with a distinctive peak occurring before spawning) further supports the idea that this behaviour may be an adaptive reproductive strategy to maximise fertilisation success. A similar behaviour; the formation of spawning aggregations, has notably been observed across diverse taxa, such as echinoderm asterids (Himmelman et al. 2008; Keesing et al. 2011), ophiurids (Himmelman et al. 2008), echinids (Simon and Levitan 2011), holoturids (Marquet et al. 2018), molluscs (for example, abalones (Shepherd 1986) and scallops (Mendo et al. 2014)), and in a wide range of fish species (see e.g., Grüss et al. (2014) and van Overzee and Rijnsdorp (2015) for reviews). The widespread occurrence of this strategy may then point towards an important selective pressure favouring aggregation in these species, reinforcing its evolutionary role in enhancing reproductive success.

### 4.2 On the Putative Role of Abiotic and Biotic Factors in *M. edulis* Aggregation Behaviour

The hypothesis that the observed synchrony between *M. edulis* aggregation and reproductive maturity plays a role in the reproductive success of this species is supported by a range of abiotic and biotic factors. First, although the observed aggregation pattern is independent of temperature seasonality, the aggregation peak occurs when the temperature reaches 12°C; in May at the onset of spawning. This is consistent with evidence that *Mytilus* sp. spawning events are triggered by an increase in temperature above 10°C–12°C (Boromtharanat et al. 1987). These suggest that temperature influences the reproductive stage and, indirectly, aggregation, further supporting the link between aggregation behaviour and reproductive maturity. Moreover, as reported by Capelle et al. (2023), the aggregation pattern observed in the present work aligns with the typical seasonal dynamics of phytoplankton in the eastern English Channel (e.g., Breton et al. 2022; Gentilhomme and Lizon 1997; Seuront et al. 2006; Seuront and Vincent 2008). Specifically, peak aggregation is synchronised with spawning and coincides with the spring phytoplankton bloom. This temporal alignment likely facilitates recruitment by ensuring that larval release occurs under optimal feeding conditions— in synchrony with the phytoplankton bloom—thereby maximising mussel reproductive success. This hypothesis is consistent with the ‘match/mismatch’ theory (Cushing 1990); the recruitment success depends on the synchrony (match) or desynchrony (mismatch) in the time between the larval production and the production of their food source, a phenomenon also previously suggested in *M. edulis* (Toupoint et al. 2012; Watson et al. 2003).

Mussels typically rely on olfactory cues to detect predators and conspecifics, hence to move and form aggregates (e.g., de Vooys 2003; Liu et al. 2011; Uguen et al. 2022, 2023). While our results showed a link between aggregation behaviour and reproductive maturity stages, the potential role of general or gender‐specific cues remains to be explored. Specifically, the observed aggregation pattern may rely on sex pheromones released by individuals with ripe gametes. In marine organisms, aggregation and mating behaviour can be stimulated by sex pheromones released by females and specifically by unfertilised mature oocytes (Boal et al. 2010; Gaudin‐Zatylny et al. 2022; Gaudron et al. 2007; Painter et al. 1998), but can also be mediated by males (Marquet et al. 2018). To gain a deeper understanding of this behaviour, further research is needed to assess the gender composition of mussel aggregations (number of males/females), the precise source of these potential pheromones, and to identify the specific molecule metabolites involved in this intricate chemical communication.

Gamete production is an exceedingly energy‐demanding process, and mussels expressed a loss of body condition following spawning (Seed and Suchanek 1992), which is consistent with the lower aggregation rate of our mussels during this period. A similar phenomenon has been observed in the *M. edulis* byssus thread quality, which exhibits a cyclical pattern linked to reproductive maturity, reaching its lowest point after the spawning season (Moeser et al. 2006). This, in addition to the low level of aggregation observed after spawning, may have significant implications, such as increased mussel dislodging risk after spawning. In aggregations, mussels rely not only on their own byssal threads but also on attachments from other mussels (de Jager et al. 2017). Therefore, mussel resistance to dislodgment is linked to both the byssus quality and neighbour quantity (de Jager et al. 2017). Given that both of these parameters reach their lowest levels after spawning, mussel collective attachment strength—the overall force required to pull a mussel out of its mussel bed—is consequently diminished. This observation is congruent with previous studies in the annual cycle of mussel collective attachment strength (Carrington 2002; Carrington et al. 2015; Lachance 2007), as opposed to individual strength (Nicastro et al. 2010; Zardi et al. 2007), which emphasised an increased risk of dislodgment during the postreproductive period. Noticeably, the postreproductive period coincides with the beginning of the storm season in the eastern English Channel (López Solano et al. 2022), hence a further increase in dislodgment risk. This may lead to mass mortality events, as commonly reported at the study site at this period of the year (i.e., around September; Seuront, pers. comm.). These events could result in an important loss of resources for both recreational and professional fishing, but also, given their bioengineering role, have ecological implications at the ecosystem level.

### 4.3 Microplastic Leachates Impair Aggregation Pattern

The aggregation pattern of *Mytilus edulis* was significantly affected by leachates from virgin polypropylene pellets, with aggregation rates being consistently high and stable from March to July and close to the maximum observed in the control group in May. These results highlight a benefit–cost aggregation threshold that could be set at *ca*. 70%, a value which, if already reached in the control group, may mask the effect of additional stressors. This hypothesis may also explain the absence of the impact of various stressors, such as predator cues and MPL, on the aggregation behaviour of specific mussel species during specific study periods (Commito et al. 2016; Seuront et al. 2021; Uguen et al. 2023).

While MPL can impair mussel chemoreception, notably by hindering the ability of mussels to recognise predator chemical cues, they do not seem to impair the ability of *M. edulis* to recognise conspecific cues (Uguen et al. 2022, 2023). In turn, as mussels aggregate more than in the control experiment, the chemical compounds in the leachate solution might stimulate the release of aggregation pheromones, potentially as a defence rather than a reproductive signal (Wertheim et al. 2005). This hypothesis is consistent with the range of harmful effects MPL cause in mussels from the cellular to the behavioural levels (Capolupo et al. 2021, 2023; Cozzolino et al. 2024; Gandara e Silva et al. 2016; Seuront et al. 2021; Uguen et al. 2022, 2023, 2024; Uguen and Cozzolino 2024). The response to MPL may involve a dynamic adjustment of aggregation behaviour in *M. edulis* until an optimum, determined by a balance between its benefits and its costs, and the mussel energy budget available for such behaviour. As the increase in aggregation behaviour following exposure to MPL is not constant throughout the year, mussels may show a ‘window of sensitivity’ to pollutants, in agreement with previous studies showing that seasonal physiological differences affect the sensitivity of species to contaminants (e.g., Dissanayake et al. 2011; Malanga et al. 2007; Ramos et al. 2014; Winner et al. 1990). This hypothesis is noticeably consistent with the energetic cost of aggregation behaviour, which may not be manageable following the spawning period to reach the *ca*. 70% optimum aggregation rate.

The observed aggregation patterns following exposure to MPL may also be related to an increase in the reproductive signal. Indeed, the main additives released by the polypropylene pellets used in this study are endocrine‐disrupting chemicals (e.g., bisphenols, phthalates and nonylphenols) that are known to lead to a range of perturbations in aquatic species, such as hormonal and reproductive impairments; see Burgos‐Aceves et al. (2021), Oehlmann et al. (2009), Zala and Penn (2004) and Zhang et al. (2021) for reviews. More specifically, it has been shown that plastic additives, notably bisphenol and phthalates, induce early spawning in *M. edulis* (Aarab et al. 2006). As a consequence, MPL may act on mussels in an advanced stage of gametogenesis (Stages 4 and 5), bringing them closer to their spawning event and potentially triggering the release of sexual aggregation pheromones. This hypothesis would explain the observed patterns of increased aggregation behaviour during this specific period, to a threshold close to control aggregation at the start of the spawning event, while highlighting the absence of a similar response during the subsequent months when mussel gonads are in immature to partial gametogenesis stages (Stages 1, 2 and 3).

Premature spawning may induce a mismatch between larvae production and optimal feeding conditions, leading to reduced recruitment (Cushing 1990). Plastic leachates have also been shown to disrupt gametes, fertilisation and larval development in various bivalves, for example, *Magallana gigas, Pinctada margaritifera, Mytilus galloprovincialis* and *Perna perna* (Capolupo et al. 2023; Gandara e Silva et al. 2016; Gardon et al. 2020; Tallec et al. 2022). Our results further suggest that MPL may act directly on the aggregation behaviour. Although this could be seen as a benefit in terms of protection against predation, wave dislodgment and thermal stress (Côté and Jelnikar 1999; Nicastro et al. 2007, 2012; Reimer and Tedengren 1997; Zardi et al. 2021), this would reduce their access to food (Nielsen and Vismann 2014). As aggregation is an energetically costly behaviour, the observed changes in aggregation rates following exposure to MPL may lead to a deficit in their energy budget and have repercussions on the species' fitness. These disturbances may have cascading effects on mussel population dynamics but also the wide range of species associated with these aggregations.

## 5 Conclusions

We showed that mussel aggregation behaviour varies seasonally in relation to their reproductive cycle. Indeed, mussel aggregation increases with gamete maturation and the proximity of the spawning event, decreasing afterwards. However, plastic leachates disrupt this seasonal aggregation pattern, primarily affecting the reproductive period by driving mussel aggregation levels to a threshold similar to the control spawning conditions. By disturbing these key functions, plastic leachates may have implications not only for mussel fitness but also at larger ecological scales, by affecting the formation of the biogenic habitats of a wide variety of species. As such, our results may be considered a stepping stone in the understanding of the effects of anthropogenic pollution on key ecological processes, and warrant the need for further work, in particular to decipher the role plastic leachates may play in mussel aggregation behaviour, that is, as a trigger of a defence signal or an amplifier of a reproductive signal.

## Author Contributions

**Marine Uguen:** conceptualization (equal), data curation (equal), formal analysis (equal), investigation (equal), methodology (equal), writing – original draft (lead), writing – review and editing (equal). **Sylvie M. Gaudron:** data curation (equal), formal analysis (equal), funding acquisition (equal), supervision (equal), validation (equal), writing – review and editing (equal). **Alexandre Rahoui‐Davoust:** data curation (equal), writing – review and editing (equal). **Valérie Lefebvre:** data curation (equal), writing – review and editing (equal). **Laurent Seuront:** conceptualization (equal), funding acquisition (equal), supervision (equal), validation (equal), writing – review and editing (equal).

## Conflicts of Interest

The authors declare no conflicts of interest.

## Supporting information


**Video S3.** Example of a time lapse with a picture taken every 0.1 s, recorded in May 2021.

**Video S4.** Example of a time lapse with a picture taken every 0.1 s, recorded in September 2021.

Appendix S1.

## Data Availability

All data supporting the results and the code used to generate the figures are freely available on Zenodo. https://doi.org/10.5281/zenodo.14034160.
