# Supplementary material for: Aggregation Behaviour as an Adaptive Reproductive Strategy in a Marine Ecosystem Engineer
Source: Ecol Evol. 2025 May 20;15(5):e71413. doi: 10.1002/ece3.71413 (PMC12092065; doi:10.1002/ece3.71413)
Supplement: Supplementary file 3 — Appendix S1. [file ECE3-15-e71413-s001.docx]

***S1. Mean and standard deviation of the sea surface temperature (°C) and Salinity (psu) for each month of 2021. Data were obtained from Coastal Coriolis data portal, for the MAREL Carnot station point (50°74’05”N, 1°56’77”E; https://data.coriolis-cotier.org)***

***S2. Additives identification protocol adapted from Uguen et al., 2023 (A) and results of the identified additives in the polypropylene pellet before (dark color) and after (light color) the 24 h incubation (B)***

**A.**

*Samples preparation*

Raw polypropylene pellets were cut using a scalpel under a binocular microscope (Olympus SZX16), to obtain fragments of ca. 500 µm². These fragments were subsequently inserted into pyrolysis quartz tubes.

*Thermodesorption method for the detection of Organic Plastic Additives (OPAs) using Py-GC-HRMS (Pyrolysis coupled to a gas chromatography and a high-resolution mass spectrometer)*

All analyses were carried out using a pyrolyser CDS Pyroprobe 6150 (CDS Analytical) followed by a GC-HRMS device (GC Trace 1310-MS Orbitrap Q exactive, Termo Fisher Scientific). With the aim to thermally desorb the chemicals additives potentially contained into the samples, they were heated into the pyrolyser at a temperature of 350 °C. Samples were subsequently separated on a GC column (Restek Rxi-5-MS capillary column, cross-linked poly 5% diphenyl-95% dimethylsiloxane, 30 m × 0.25 mm (i.d.) × 0.25 µm film thickness), with a split ratio of 1:5. The acquisition was performed on full-scan (FS) mode (m/z = 30.00000–600.00000). The resulting chromatograms were analysed using Xcalibur and TraceFinder softwares to identify OPAs among a selection of additives including plasticisers, flame retardants, antioxidants and UVs stabilisers. The respective additives were identified on the basis of the retention times, m/z, and specific ions after a comparison with the chromatograms of the respective standard solutions.

*Identified additives*

- 3 brominated flame retardants, *i.e.* 2,2',4,4',5,5'-Hexabromodiphenyl ether (BDE-153); 2,2',4,4',5,6'-Hexabromodiphenyl ether (BDE-154) and 2,2',3,4,4',5',6-Heptabromodiphenyl ether (BDE-183);
- 6 phosphorus flame retardants, *i.e.* Tributyl Phosphate (TBP); Triethyl Phosphate (TEP); Tris(2-Chloroethyl)Phosphate (TEPP); Tris(2-Chloroisopropyl)Phosphate (TCPP); Tris(1,3-Dichloro-2-Propyl)Phosphate (TDCPP) and Triphenyl Phosphate (TPhP)
- 5 antioxidants, *i.e.* Butylated hydroxytoluene (BHT); Bisphenol F (BPF); Bisphenol S (BPS); Nonylphenol (NPs) and Nonylphenol monoethoxylate (NP10E)
- 13 plasticisers, *i.e.* Dimethyl phthalates (DMP); Diethyl phthalate (DEP); Di-n-butyl phthalate (DBP); Di-allyl phthalate (DAIP); Diisobutyl phthalate (DIBP); Benzylbutyl phthalates (BBP); Diisoheptyl phthalate, (DIHP); Bis(2-Ethylhexyl) phthalate (DEHP); Diisononyl phthalate (DINP); Diisodecyl phthalate (DIDP); Diisononyl hexahydrophthalate (DINCH); Di-n-octyl phthalate (DIOP); Bis-2-Ethylhexyl Adipate (DEHA)

**B.**


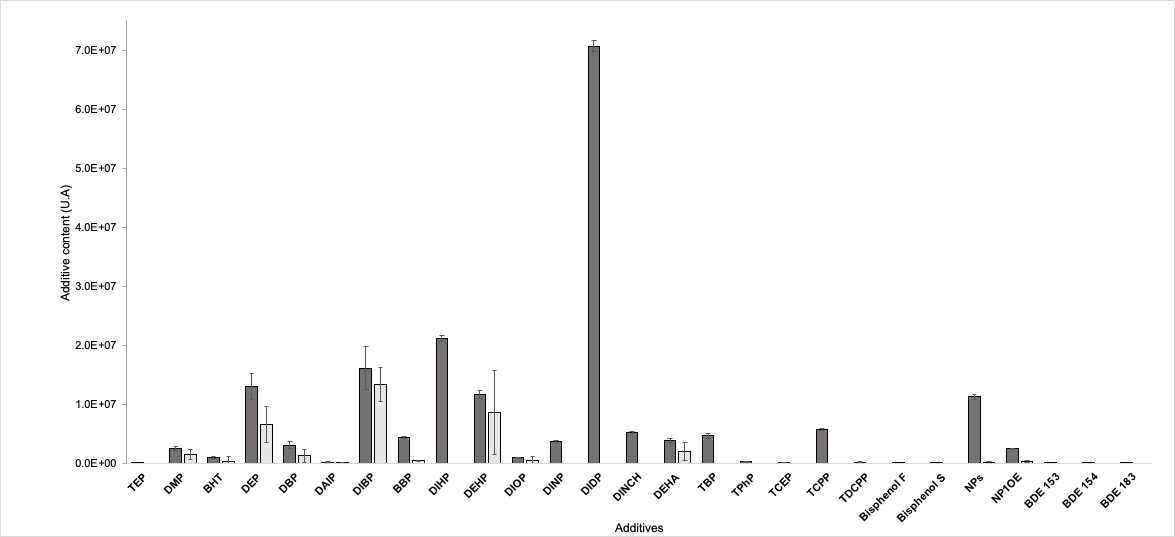


***S3. Example of a time lapse with a picture every 0.1 second, in May 2021***

***
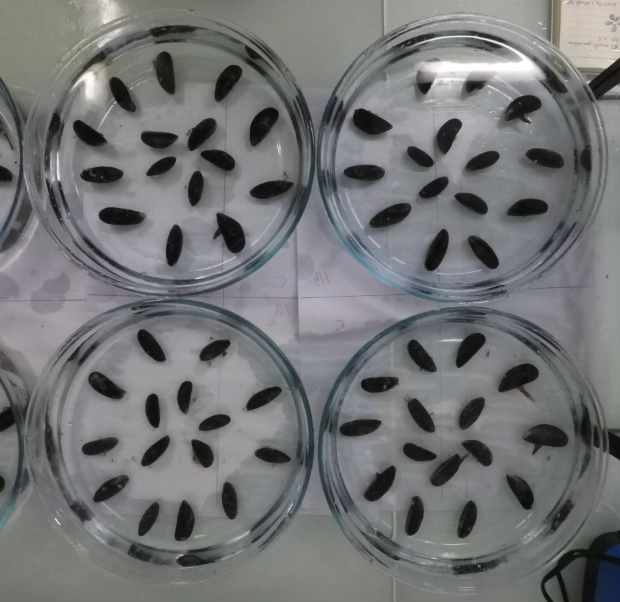
***

***S4. Example of a time lapse with a picture every 0.1 second, in September 2021***

*
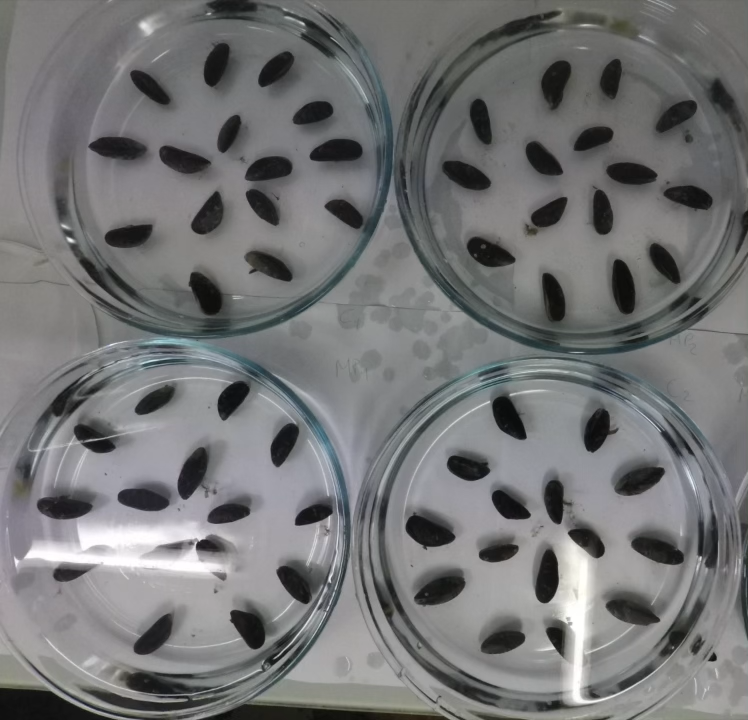
*

***S5. Determination of stages of reproduction of both sex***

The sexual maturity of all 20 specimens could not be achieved and a total of 105 specimens were assessed from March to December 2021, composed of 35 males, 66 females and 4 undifferentiated individuals, with at least 7 individuals by month (min= 7; max= 11; for details see Table S1). Five main stages are described following different criteria from previous studies (Chiperfield, 1951; Seed, 1969; Auffret at al., 2003; for details see Fig. 1, 2). For females, the mean oocytes Feret diameter was used in a final decision to attribute the stage of reproduction. The frequency of the different reproductive stages of *Mytilus edulis* sample in 2021 was thus obtained (Fig. 3).

*Table 1:* details about the individuals used to assessed the histological stage, *i.e.,* sex, stages, total number and measured mean sexual stage.

*Ranking and criteria for female (Fig. 1)*

**Stage I**: **Resting stage** **(Fig.1A&B)**

This is a transition stage where females have spawned all their oocytes, and the gonad is in a resting stage. Some acinus can be seen still with few oocytes but those are atretic being lysed with some anomalies in their membrane or in their cytoplasm. Most of acini are empty. Some pink coloration highlights special cells, with the adipogranular cells that accumulate protein and lipid, and the vesicular cells that are translucent that accumulate glycogen.

**Stage II: Start of oogenesis (Fig.1C&D)**

This stage is characterised by the proliferation of oogonia by mitosis, where oogonia are attached to the follicle membrane. There is a highly presence of reserve cells between follicles.

**Stage III: Vitellogenesis (Fig.1E&F)**

Vitellogenic oocytes are increasing in size but are still anchored into the follicle membrane. They look like pedunculated and a nucleolus is visible. The number of storage reserve is decreasing.

**Stage IV: Maturation (Fig.1G&H)**

Oocytes are fully-mature and free within the follicle, ready to be released. They have attained their optimal size (mean Feret oocyte diameter). Follicles fulfilled the gonad with the disappearance of reserve cells.

**Stage V: Spawning stage (Fig.1I&J)**

Most of follicles are empty but the spawning is partial and there are still follicles with oocytes at different stages (vitellogenic and mature oocytes). There is a reappearance of reserve cells.

*Figure 1:* Histological sections through female gonad of *Mytilus edulis* at various stages of reproduction stained with Haematoxylin and Eosin. Resting stage (undifferentiated; A & B). Start of oogenesis (early development; C & D). Vitellogenesis (late development; E & F). Maturation (ripe; G & H). Spawning stage (spent; I & J). (ao) atretic oocytes; (adp) adipogranular cells; (vc) vesicular cells; (oo) oogonia; (vo) vitellogenic oocyte; (mo) mature oocyte. Scale bars are 100 µm for A, C, E, G & I. Scale bars are 20 µm for B, D, F, H & J.

*Ranking and criteria for male (Fig. 2)*

**Stage I**: **Resting stage (Fig.2A&B)**

This is a transition stage where males have spawned by releasing all the spermatozoids. The gonad is in a resting stage. Few germ cells are present in purple. Some pink coloration highlights special cells, with the adipogranular cells that accumulate protein and lipid, and the vesicular cells that are translucent that accumulate glycogen.

**Stage II: Start of spermatogenesis (Fig.2C&D)**

This stage is characterised by the proliferation of spermatogonia by mitosis, with some differentiation towards the centre of the follicle of spermatocyte. Islands of germinal tissue appeared at the base of the acinus like a branch. There is a highly presence of reserve cells between follicles.

**Stage III: Growth and maturation (Fig.2E&F)**

The number of follicles with male gametes has increased with the purple coloration starting to dominate in the histological section. Different stages of male gametes are visible (spermatogonia, spermatocytes and spermatozoids) with the dominance of spermatocytes. The number of storage reserve is decreasing.

**Stage IV: Fully ripe (Fig.2G&H)**

The pink coloration within the gonad has disappeared. The gonad is fulfilled with follicles dominated by spermatozoid arranged in rosettes with their flagella in the lumen of the follicle.

**Stage V: Partial spawning stage (Fig.2I&J)**

Some follicles are devoid of male gametes indicating a partial spawning event, but there are still follicles in ripe stages with spermatozoids. However, between these ripe follicles there is the reappearance of reserve cells.

*Figure 2:* Histological sections of male gonad of *Mytilus edulis* at various stages of reproduction stained with Haematoxylin and Eosin. Resting stage (undifferentiated; A & B). Start of spermatogenesis (early development; C & D). Growth and maturation (late development; E & F). Fully ripe (ripe; G & H). Partial spawning stage (spent; I & J). (adp) adipogranular cells; (vc) vesicular cells; (gc) germinal cells; (spg) spermatogonia; (spc) spermatocyte; (spz) spermatozoid. Scale bars are 100 µm for A, C, E, G & I. Scale bars are 20 µm for B & D. Scale bars are 10 µm for F, H & J.

*Figure 3:* Frequency of the different reproductive stages of *Mytilus edulis* sample in 2021 from a rocky intertidal reef (Pointe aux Oies, Wimereux; 50°47’08.3"N, 1°36’03.9"E)

***S6.*** ***Results of Conover post-hoc test with Holm’s correction for Control data. P-values are rounded to 3 decimal places.***

***
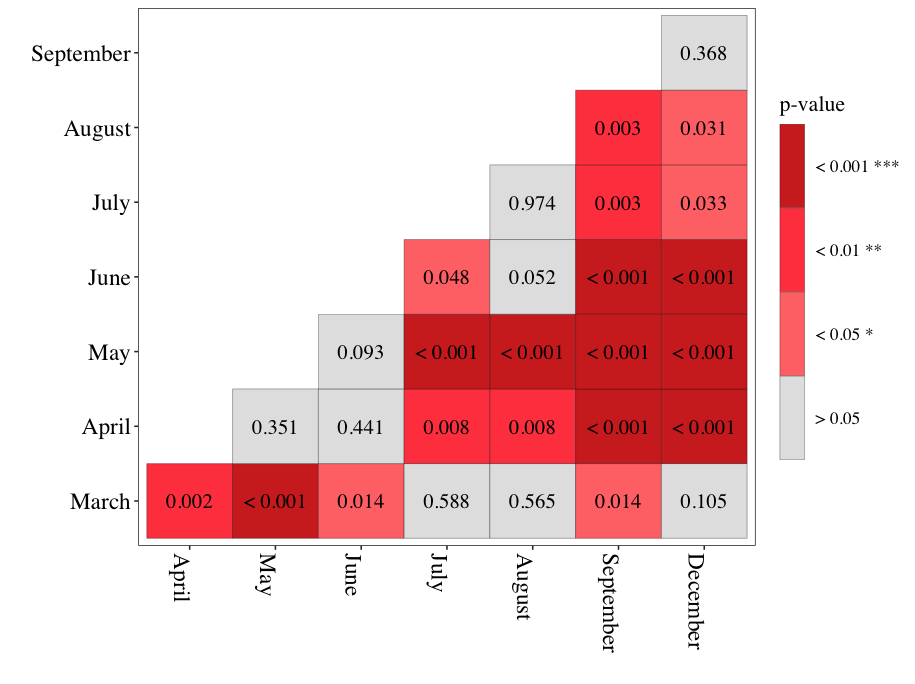
***

***S7. Results of Conover post-hoc test with Holm’s correction for Microplastic leachate data. P-values are rounded to 3 decimal places.***


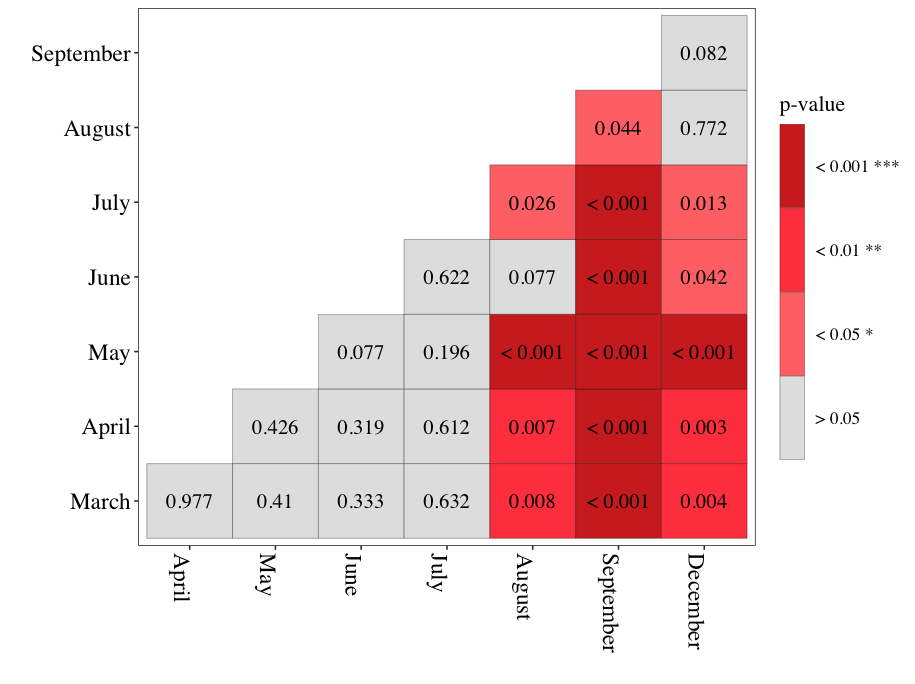


***S8. Measured and estimated values for the aggregation rate (%) for control (grey) and microplastic leachates data (black) and their respective correlation coefficient (R^2^). The dashed line is the first bissectrix, i.e. Measured values = Estimated values. Estimated values are determined by using the following equation: Agg(µ) = 31.04 ⋅ 𝑠𝑖𝑛(1.63 ⋅ µ - 11.72) + 53.28; with µ the mean reproductive stage.*** ****** = p-value < 0.001.***

***
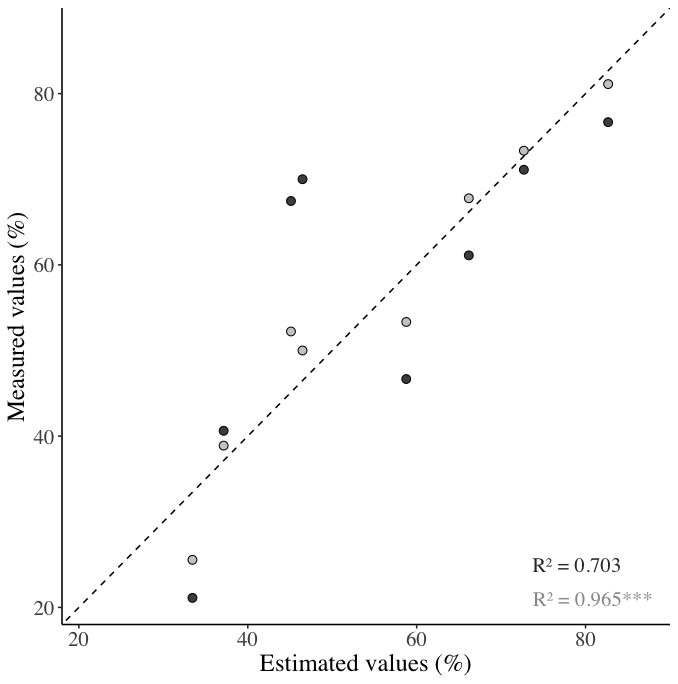
***
